# DNA damage induces nuclear translocation of parkin

**DOI:** 10.1186/1423-0127-16-67

**Published:** 2009-07-17

**Authors:** Shyan-Yuan Kao

**Affiliations:** 1 Eaton Peabody Laboratory, Massachusetts Eye and Ear Infirmary, 243 Charles Street, Boston MA 02114. USA

## Abstract

Parkinson's disease (PD) is the second most common form of human degenerative disorder. Mutation of *parkin *is one of the most prevalent causes of autosomal recessive PD. Parkin is an E3 ubiquitin ligase that acts on a variety of substrates, resulting in polyubiquitination and degradation by the proteasome or monoubiquitination and regulation of biological activity. However, the cellular functions of parkin that relate to its pathological involvement in PD are not well understood. Here I show that parkin translocates into nucleus upon DNA damage. Nuclear translocation of parkin appears to be required to promote DNA repair. These findings suggest that DNA damage induces nuclear translocation of parkin leading to the PCNA interaction and possibly other nuclear proteins involved in DNA repair. These results suggest that parkin promotes DNA repair and protects against genotoxicity, and implicate DNA damage as a potential pathogenic mechanism in parkinsonism.

## Introduction

Parkin was first isolated as the candidate gene of autosomal recessive juvenile Parkinsonism (AR-JP) [1]. Although many types of mutations, such as point mutations, truncations and/or splicing variants of *parkin*, are found in Parkinson's disease (PD) patients [2], the mechanism of how parkin is involved in PD remains elusive. In addition to its association with PD, parkin might be associated with carcinogenesis and may be a tumor suppressor [3]. Abnormal parkin transcripts have been detected in a number of different tumor cell types, including lung, cervical, pancreatic, and kidney tumors [3].

Parkin functions as an E3 ubiquitin protein ligase [1]. Based on its structure, parkin may act as a docking station bringing substrate proteins to an E2 ubiquitin conjugating enzyme for ubiquitination, thus leading to their degradation [4]. Several parkin interacting proteins have been identified, including CDCrel-1 [5], glycosylated α-synuclein [6], synphilin-1 [7], PAEL-R [8], cyclin E [9], aminoacyl-tRNA synthetase cofactor p38 [10], and a PDZ containing protein CASK/Lin2 [11]. However, the mechanism by which parkin mutations cause PD remains to be determined.

Accelerated DNA damage occurs during the aging of the human brain [12]. Furthermore, several studies suggest that DNA damage is involved in neurodegenerative processes [13,14]. For example, the level of the DNA damage marker, 8-hydroxyguanine is increased in genomic DNA in PD substantia nigra compared to non-PD controls [15]. In addition, somatic mitochondrial DNA mutations in cortex and substantia nigra are increased in PD [16]. Furthermore, it has been proposed that oxidative stress induced DNA damage could be an important risk factor for PD [17,18].

These observations prompted us to examine genes associated with PD for a role in DNA damage response. The current studies provide evidence that DNA damage promotes nuclear translocation of parkin. These findings provide a potential interface between normal aging associated DNA damage and pathologic associated with neurodegenerative disease, and is consistent with the very early onset of PD associated with parkin mutations.

## Materials and methods

### Antibodies

Rabbit anti-Parkin antibodies and rabbit anti-β-actin antibodies were purchased from Cell Signaling. Mouse anti-PCNA antibodies were from Santa Cruz Biotechnology. Goat anti-8-OHdG antibodies and mouse anti-lamin B antibodies were obtained from Chemicon, and mouse anti-CPD was from MBL.

### Cells and tissues

SH-SY5Y human neuroblastoma cells and HeLa cells were grown in DMEM supplemented with 10% fetal bovine serum. Parkin and parkin mutant (Del 3–4) stable cells were established by transfecting pcDNA3.1(-)-Parkin or pcDNA3.1(-)-Parkin-Del 3–4 into SY5Y cells, and selected by geneticin. Post-mortem human cortical samples were described previously [12].

### Chromatin immunoprecipitation

The association of parkin with calmodulin 1 promoter was assayed by chromatin immunoprecipitation (ChIP) using the ChIP assay protocol (Upstate) with some modifications. Brain tissue samples were prepared as described before [12]. HeLa cells transfected with parkin and UV damaged or undamaged calmodulin promoter constructs was harvested according to ChIP assay protocol. The amount of DNA in the resulting cell lysates was quantified by measuring absorption at 260 nm, and then adjusted to 100 μg/μl. 200 μg supernatant was diluted 10-fold in 2 ml ChIP dilution buffer (0.01% SDS, 1.1% Triton X-100, 1.2 mM EDTA, 16.7 mM Tris-HCl, pH 8.1, 167 mM NaCl, and protease inhibitors), and precleared twice with BSA-blocked Protein L Agarose (Pierce) (2 × 100 μg, 2 × 30 min at 4°C). The beads were centrifuged and the supernatant was divided into 4 × 500 μl aliquots for immunoprecipitation, input DNA, and the IgG control. Primary antibody was added and incubated at 4°C overnight. A rabbit anti-parkin polyclonal antibody (Cell signaling) was used for immunoprecipitation of Parkin, and ChromPure rabbit IgG (Jackson ImmunoResaerch) was used for the IgG control. 30/μl of BSA-blocked Protein L Agarose was then added and incubated at 4°C with rotation. The beads were then centrifuged and washed once with a low salt immune complex buffer (Upstate), twice with a high salt wash buffer, once with a LiCl wash buffer (Upstate), and twice in TE buffer (10 mM Tris-HCl, 1 mM EDTA, pH 8.0). The washed agarose beads were eluted with 2 × 250 μl freshly prepared elution buffer (1% SDS, 0.1 mM NaHCO_3_). DNA crosslinking was reversed by adding 5 M NaCl and heating at 65°C for 4 hrs. Protein was removed by incubation with 20 mg/ml proteinase K in 10 μg EDTA/40 mM Tris-HCl, pH 6.5 for 1 hr at 45°C. De-crosslinked DNA was then isolated by phenol/chloroform extraction and ethanol precipitation. The precipitated DNA was washed with 70% ethanol, air dried and dissolved in ddH_2_O for PCR.

### Induction of DNA damage

UV-irradiation was performed by irradiating SH-SY5Y cells with UV-C light (254 nm, 60 J/m^2^) using a Stratalinker (Stratagene). Oxidative DNA damage was performed on SH-SY5Y cells treated with H_2_O_2 _(300 μM) for overnight. Double strand break DNA damage was induced by treatment with 50 μM etoposide (Calbiochem) for overnight.

### Immunohistochemical staining of DNA cyclopyrimidine dimers (CPD)

Immunohistochemical staining of CPD was performed after irradiating SH-SY5Y cells with 100 J/M^2 ^of 254 nm UV light using a microfliter (Millipore). Cells were then fixed 1 hr or 12 hr later with 4% paraformaldehyde, permeabilized for 15 min in 0.4% Triton X-100 (Sigma) in PBS, and the DNA was denatured in 2 M HCl for 30 min at room temperature. After blocking with FBS, cells were incubated with anti-CPD antibody (1:3000) for 30 min. The sections were then incubated with Cy3-conjugated anti-goat IgG (Jackson ImmunoResearch) at room temperature for 30 min and then with Hoechst dye for 5 min. The sections were analyzed by fluorescence transmission or confocal microscopy.

### Host cell reactivation assays

The host cell reactivation assay (HCR) was performed as described previously [19,20]. A pTK-Renilla reporter plasmid was damaged by exposure to UV-C light (254 nm) at 250 J/m^2 ^*in vitro*, which markedly reduces its expression. SH-SY5Y cells were then transfected with UV-irradiated or control non-irradiated pTK-Renilla expression vectors. Each plate was co-transfected with a control firefly luciferase vector (pGL3-basic) to control for transfection efficiency. Cells lysis was performed 24 hours after transfection, renilla and firefly luciferase activities were measured, and renilla luciferase activity was normalized to the firefly luciferase activity in the same plate. Renilla luciferase activity of the control undamaged plasmid was normalized to 100%, and compared with the expression of the UV-irradiated reporter plasmid in the same cell type.

## Results

### DNA damage and nuclear translocation of parkin

**Figure 1:**
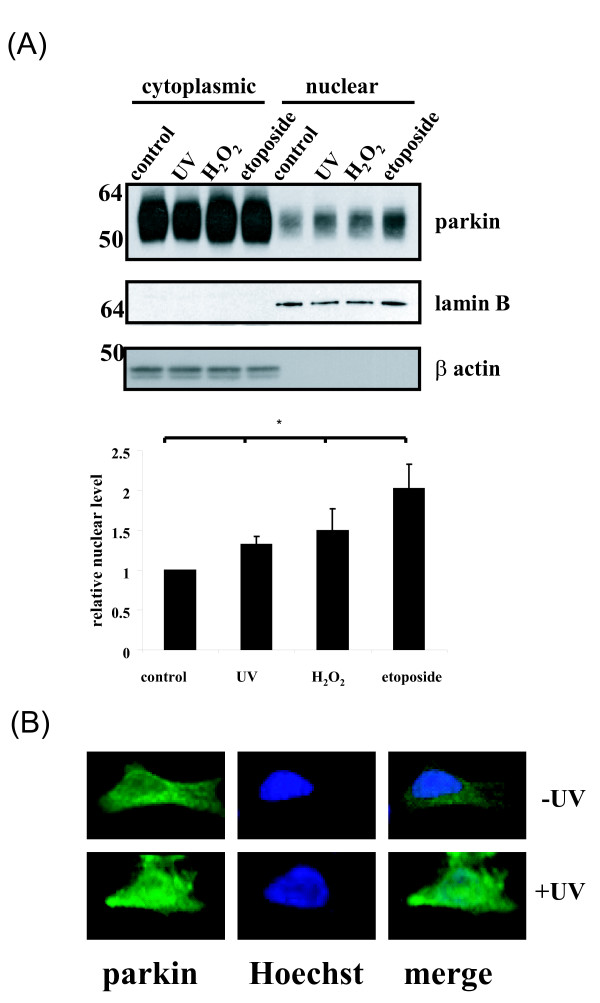
**DNA damage and nuclear translocation of parkin**. (A) DNA induces nuclear translocation of parkin. SH-SY5Y cells stably transfected with parkin were UV-irradiated (60 J/m^2^), or treated with H_2_O_2 _(300 μM) or etoposide (50 μM) to induce DNA damage, followed by the isolation of nuclear and cytoplasmic fractions and resolution of parkin by immunoblotting. Note that each DNA damaging agent induced an increase in the absolute and relative levels of parkin in the nucleus. Absence of cross-contamination of the fractions was confirmed by immunoblotting for lamin B, a nuclear marker, and β-actin, a cytoplasmic marker. Quantitation of the relative nuclear ratio of parkin is shown, and represents the mean ± S.D., n = 3. *P < 0.05 relative to control by ANOVA with post-hoc Student Neumann-Kiels tests. (B) Nuclear translocation of parkin in SH-SY5Y cells was confirmed by immunofluorescence assays. SH-SY5Y cells stably over-expressing parkin were UV-irradiated (60 J/m^2^) or mock-treated. Two hours later the cellular localization of parkin was determined by immunofluorescent microscopy. Green: parkin. Blue: Hoechst staining of nucleus.

I first asked if parkin is translocated to the nucleus upon DNA damage. To assess nuclear translocation, SH-SY5Y cells stably over-expressing parkin were analyzed by subcellular fractionation after exposure to agents that induce DNA damage. Cell lysates were separated into nuclear and cytoplasmic fractions, and parkin was resolved by immunoblotting (Fig 1, top). The ratio of nuclear to cytoplasmic parkin was significantly increased after UV irradiation, treatment with H_2_O_2_, and treatment with etoposide, which selectively induces DNA double strand breaks (Fig 1A, bottom). Absence of cross-contamination of the fractions was confirmed by immunoblotting for lamin B, a nuclear marker, and β-actin, a cytoplasmic marker. Thus, a variety of different DNA damaging insults can induce the nuclear translocation of parkin.

The nuclear translocation of parkin was confirmed by immunofluorescence assays. SH-SY5Y cells stably over-expressing parkin were irradiated by UV-C light (60 J/M^2^). The nuclear translocation of parkin was assayed 2 hr after treatment. Parkin expression was mainly cytoplasmic without UV irradiation (Fig 1B, top), while the expression of parkin in the nucleus was greatly increased after UV irradiation (Fig 1B, bottom). These results confirmed the nuclear translocation of parkin after DNA damage.

### Parkin associates with damaged DNA

**Figure 2:**
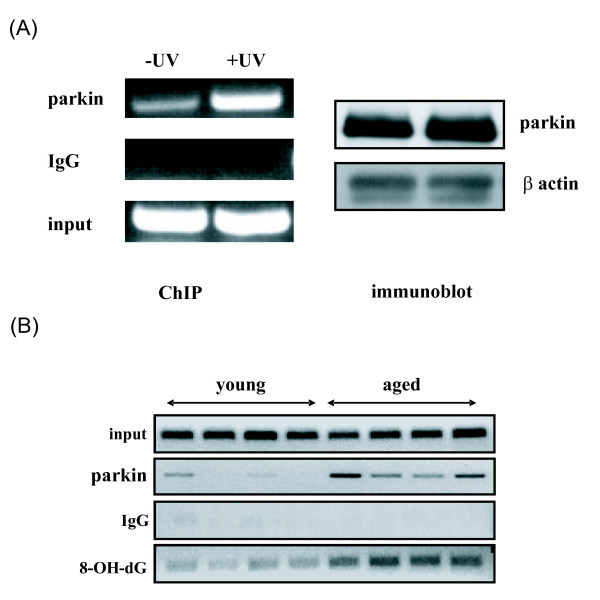
**Parkin interacts with damaged DNA**. (A) Parkin was co-transfected in HeLa cells with a calmodulin 1 promoter construct under control conditions (-UV) or after the calmodulin construct was damaged by UV irradiation *in vitro *(+UV)(100 J/M^2^). To determine whether parkin interacts with damaged DNA, parkin was immunoprecipitated followed by PCR amplification of the calmodulin promoter construct. Note the markedly increased co-precipitation of parkin with DNA damaged by UV irradiation. Input DNA, non-specific IgG controls, and parkin, β-actin immunoblots are shown. (B) Parkin binds to chromatin in the aging human brain. ChIP assays were performed on postmortem human cortical samples with anti-parkin or anti-8-oxoguanine followed by PCR amplification of a calmodulin-1 promoter sequence that is damaged in human cortical samples from aged and young adult individuals. Note increased binding of parkin to chromatin from some aged cortical samples (≥ 73 years old) relative to young adult samples (<40 years old), which correlates with increased 8-oxoguanine content. Input and non-specific IgG ChIP controls are shown.

To further explore whether parkin is involved in DNA damage repair, I examined whether parkin associated with damaged DNA. A human calmodulin 1 promoter construct was either UV irradiated *in vitro *or untreated, and then was co-transfected with a parkin construct into HeLa cells. The human calmodulin 1 promoter was chosen because it was preferentially damaged in human ageing brain [12], and the HeLa cell line was used because HeLa cells contain a *parkin *gene deletion [21] and may not express functional parkin protein [22,23]. ChIP assays were performed to determine the association between parkin and DNA. There was a weak interaction between parkin and untreated calmodulin 1 promoter (Fig. 2A), however such interaction was significantly increased when DNA was damaged by UV irradiation (Fig. 2A).

To demonstrate that parkin associated with damage DNA also occurred in human brain tissues with endogenous damaged DNA, I next investigated the association between parkin and the endogenous calmodulin 1 promoter in normal human brain tissues. After analyzing tissue samples from 4 young brains (<40-year-old) and 4 old brains (>70-year-old), ChIP assays showed a much stronger association between parkin and calmodulin 1 promoter in the aged brain tissues than that in young brain tissues (Fig. 2B). Since there were higher levels of DNA damage in aged human brains as shown by the presence of high level of 8-OHdG in calmodulin 1 promoter (Fig. 2B, bottom), these results suggest that parkin is associated with damaged DNA *in vivo*.

### Parkin reduces DNA damage induced by UV irradiation

**Figure 3:**
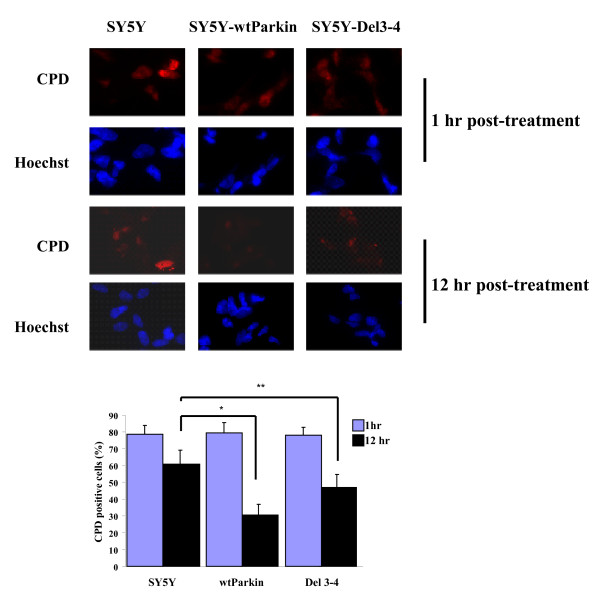
**Parkin reduces DNA damage induced by UV irradiation**. Stably SH-SY5Y cell lines expressing wild-type parkin, the Del3–4 parkin mutant, or the pcDNA control vector were UV-irradiated (100 J/M^2^) and then assessed for DNA damage by immunocytochemical staining for cyclopyrimidine dimers (CPD) (red). Nuclei were stained with Hoechst dye. All three cell lines show similar robust CPD staining 1 hr after UV irradiation. Decreased DNA damage in SH-SY5Y cells expressing parkin, but not the pcDNA control and Del3–4 parkin mutant was detected. Quantitation of CPD positive cells in three cell lines after UV irradiation is shown, and represents the mean ± S.D., n = 5. *P < 0.05, **P > 0.05 relative to control by ANOVA with post-hoc Student Neumann-Kiels tests.

Since parkin associates with damaged DNA complex, this observation prompted me to ask whether parkin is involved in the process of DNA repair. To address this question, DNA damage was induced by UV irradiation of SH-SY5Y cells that stably over-express wild-type parkin or control expression plasmids. DNA damage was examined by immunofuorescence microscopy with an antibody to cyclobutane pyrimidine dimers (CPD), a major DNA adduct induced by UV irradiation. UV irradiation induced substantial DNA damage in all three cell lines as indicated by robust nuclear immunoreactivity for CPD (Fig. 3, top, 1 hr post-treatment). This damage was markedly reduced by over-expression of wild-type parkin after 12 hr (Fig. 3, top, 12 hr post-treatment). In contrast, over-expression of a parkin exon 3–4 deletion construct, which is associated with autosomal recessive PD, did not appear to reduce UV-induced CPD immunoreactivity (Fig 3, top, 12 hr post-treatment, and bottom).

### Nuclear localized parkin promotes DNA excision repair

**Figure 4:**
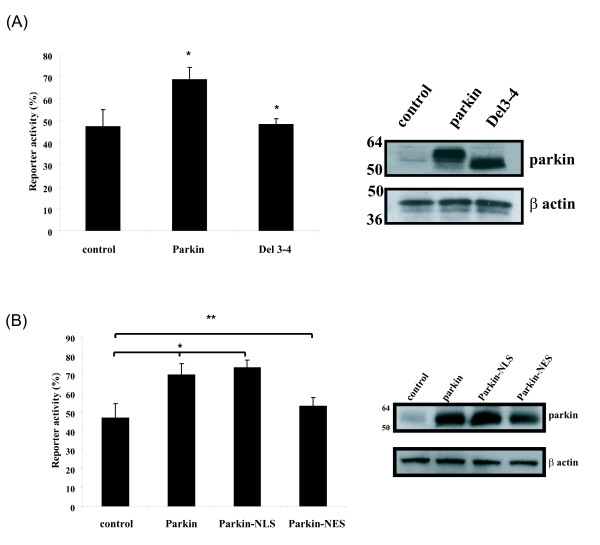
**Nuclear localized parkin promotes DNA excision repair**. (A) HCR assays showed Parkin facilitates DNA repair of UV induced DNA lesions in SH-SY5Y cells (pTK-Renilla irradiated by UV-C at 250 J/m^2^). Values are expressed relative to the transfected undamaged reporter, and represent the mean ± s.d.; *n *= 3. Asterisks indicate *P *< 0.05 relative to control by ANOVA with post-hoc Student Neumann-Kiels tests. Western blot analysis showed the expression of wild type parkin and parkin Del3–4 mutant. (B) HCR assays showed the ability of wild type Parkin, Parkin-NLS, and Parkin-NES to regulate DNA repair activities. *n *= 3. * *P *< 0.05, ** *P *> 0.05 relative to control by ANOVA with post-hoc Student Neumann-Kiels tests. Western blot analysis showed the expression of wild type parkin, parkin-NLS, and parkin-NES.

In a previous study, I have shown that parkin interacts with proliferating cell nuclear antigen (PCNA), and promotes DNA excision repair [24]. I next tested whether nuclear translocation is important for the promotion of DNA excision repair by parkin. Host cell reaction assays were used to determine the ability of parkin to promote nucleotide excision repair. I found that the over-expression of parkin could facilitate DNA repair. In the HCR assays, UV damaged or control renilla reporter constructs were transfected into SH-SY5Y cells together with parkin expression constructs, parkin Del 3–4, or vector backbone pcDNA3.1. Wild type Parkin transfected cells had approximate 30% higher cellular DNA repair activity than mock-transfected cells (Fig. 4A), while the Parkin Del 3–4 mutant had little effect on DNA repair. This result indicates that Parkin facilitates DNA repair induced by UV irradiation.

Parkin is shown to be localized dominantly cytoplasm with some fraction of parkin expressed in mitochondria and nucleus. To further explore the importance of nuclear translocation and regulation of DNA repair by parkin, several parkin expressing plasmids were constructed. The nuclear localization signal (NLS) of SV40 large T antigen [25] and the nuclear export signal (NES) of HIV Rev [26] were inserted upstream of parkin coding sequences respectively so that parkin can be localized exclusively in either cell nucleus (Parkin-NLS) or cytoplasm (Parkin-NES) in SH-SY5Y cells. I next tested the ability of these constructs to regulate DNA repair activity. Using HCR assays, wild type Parkin and Parkin-NLS facilitate DNA repair more than 30% compared to control cells (Fig. 4B), while Parkin-NES had little effect on DNA repair, indicating that nuclear localized parkin is indispensable for the regulation of DNA repair activity.

## Discussion

These findings suggest that a broad range of DNA damaging agents induce nuclear translocation of parkin, including hydrogen peroxide, which induces oxidative base damage, UV irradiation, which induces pyrimidine dimer formation, and etoposide, which induces DNA double strand breaks. It has been shown that parkin-deficient mice show increased 8-oxoguanine in cerebral cortex, and parkin promotes both base and nucleotide excision repair in cultured cells [24]. In addition, parkin associates with PCNA, which mediates DNA excision repair. Thus, parkin may be involved in multiple DNA repair systems, including base and nucleotide excision repair and double strand break repair.

Nuclear translocation of parkin appears to be required, as parkin fused to a nuclear export signal is unable to promote DNA repair. These findings suggest that DNA damage induces nuclear translocation of parkin leading to the PCNA interaction and possibly other nuclear proteins involved in DNA repair. These results also replicate the findings of a previous report which showed that oxidative stress can induce nuclear translocation of parkin [27]. The mechanism of nuclear translocation remains to be determined, as parkin does not contain a canonical nuclear localization signal. Parkin could potentially translocate in a complex with PCNA or other DNA repair proteins triggered through DNA damage-mediated signaling. In addition to PCNA, a previous report using yeast two hybrid assays identified a parkin interaction with rad1, a component of the rad9-hus1-rad1 protein complex involved in DNA repair [28,29]. These results suggest that parkin promotes DNA repair and protects against genotoxicity, and implicate DNA damage as a potential pathogenic mechanism in parkinsonism.

## Competing interests

The author declares that they have no competing interests.

## Authors' contributions

SYK designed and performed experiments, and prepared the manuscript.
